# Interaction of clozapine with metformin in a schizophrenia rat model

**DOI:** 10.1038/s41598-021-96478-2

**Published:** 2021-08-19

**Authors:** G. Horvath, G. Kis, G. Kekesi, A. Büki, L. G. Adlan, E. Szűcs, H. El Heni, S. Benyhe

**Affiliations:** 1 Department of Physiology, Faculty of Medicine, University of Szeged, Dóm tér 10, 6720 Szeged, Hungary; 2 Institute of Biochemistry, ELKH Biological Research Center, 6726 Szeged, Hungary

**Keywords:** Molecular biology, Neuroscience, Physiology, Diseases, Neurology, Pathogenesis, Signs and symptoms

## Abstract

The low efficacy of antipsychotic drugs (e.g., clozapine) for negative symptoms and cognitive impairment has led to the introduction of adjuvant therapies. Because previous data suggest the procognitive potential of the antidiabetic drug metformin, this study aimed to assess the effects of chronic clozapine and metformin oral administration (alone and in combination) on locomotor and exploratory activities and cognitive function in a reward-based test in control and a schizophrenia-like animal model (Wisket rats). As impaired dopamine D1 receptor (D_1_R) function might play a role in the cognitive dysfunctions observed in patients with schizophrenia, the second goal of this study was to determine the brain-region-specific D_1_R-mediated signaling, ligand binding, and mRNA expression. None of the treatments affected the behavior of the control animals significantly; however, the combination treatment enhanced D_1_R binding and activation in the cerebral cortex. The Wisket rats exhibited impaired motivation, attention, and cognitive function, as well as a lower level of cortical D_1_R binding, signaling, and gene expression. Clozapine caused further deterioration of the behavioral parameters, without a significant effect on the D_1_R system. Metformin blunted the clozapine-induced impairments, and, similarly to that observed in the control animals, increased the functional activity of D_1_R. This study highlights the beneficial effects of metformin (at the behavioral and cellular levels) in blunting clozapine-induced adverse effects.

## Introduction

Schizophrenia is a chronic and highly impairing neuropsychiatric disease that affects around 1% of the human population. In addition to the positive (e.g., delusions and hallucinations) and negative (e.g., asociality, avolition, and amotivation) symptoms, cognitive deficits (impaired attention, learning, and memory functions) are a hallmark of this disease^1,2^. Both first-generation (or typical; e.g., haloperidol) and second-generation (or atypical; e.g., clozapine [CZP], olanzapine, and risperidone) antipsychotics primarily relieve the positive symptoms, whereas the negative symptoms and cognitive deficits remain largely unaffected by these treatments^3^. Therefore, several types of adjuvant therapies (e.g., physical exercise, cognitive training, and procognitive or antidepressant drugs) have been proposed in the treatment of patients with schizophrenia^4,5^. Metformin (MTF) is a safe and effective agent that is widely applied for the management of patients with type 2 diabetes mellitus; however, it is also used in the treatment and prevention of the second-generation antipsychotic-induced impairments in lipid and glucose metabolism and weight gain^5,6^. Several reports have suggested that MTF improves cognition in different conditions, including Parkinson’s and Alzheimer’s diseases^7,8^; however, no data are available regarding its effects on cognitive function in patients with schizophrenia.

Translational research depends on the relevance of animal models that replicate the human disease and investigate the mechanisms of action of different potentially beneficial drugs for the treatment of schizophrenia. To provide high validity for this disease, a “multiple hit” rat model, termed Wisket, was developed by combining developmental (post-weaning social isolation), pharmacological (treatment with the NMDA receptor antagonist ketamine), and genetic (selective breeding based on behavioral phenotype for more than 30 generations) manipulations. Wisket animals exhibit a wide range of disturbances, including decreased pain sensitivity, sensory gating, cognitive impairments, and altered dopamine 2 receptor (D_2_R) functions^9–12^. Although not included in the diagnostic criteria for schizophrenia, several data report decreased pain sensitivity in this disease, and its animal models^13^. Therefore, we supposed that the evaluation of pain sensitivity might also be an important sign of behavioral impairments.

Both human and animal studies have reported conflicting results regarding the effect of CZP on cognitive functions^14–18^, and only a few studies have suggested beneficial effects of MTF in schizophrenia rodent models^19,20^. Therefore, the first goal of this study was to determine the effects of chronic treatment (4 weeks) with CZP, MTF, and their combination (MTF_CZP) on several parameters related to the locomotor and exploratory activities and cognitive functions of control (Wistar) and model (Wisket) rats in a reward-based learning test (Ambitus).

Clinical and preclinical studies reported significant roles of the dopamine D_1_ receptors (D_1_Rs) in the impaired cognitive disturbance of patients with schizophrenia^21–23^. Although the mechanism of action of MTF is not fully understood, it seems that both AMP-activated protein kinase (AMPK)-dependent and -independent pathways might be responsible for its neuroprotective activity^7^. No data are available regarding the effects of MTF on D_1_Rs; thus, the second aim of our study was to characterize the D_1_R-mediated signaling, radioligand binding, and mRNA expression in control and Wisket animals in different brain structures, and the potential effect of CZP, MTF, and their combination on these parameters. D_1_R binding, signaling, and mRNA expression patterns were determined in the cerebral cortex (CTX). Moreover, binding and signaling experiments were also performed in the olfactory bulb (OB), brainstem, and diencephalon. Finally, D_1_R mRNA expression was detected in the prefrontal cortex (PFC), striatum, cerebellum, and hippocampus.

## Results

In agreement with our recent studies^9,10^, Wisket rats showed decreased pain sensitivity (F_(1,74)_ = 81.46; *P* < 0.001), sensory gating (F_(1,74)_ = 5.14; *P* < 0.05), locomotor activity (F_(1,74)_ = 4.69; *P* < 0.05), exploratory activity (F_(1,74)_ = 24.05; *P* < 0.001), and learning capacity (F_(1,74)_ = 14.89; *P* < 0.001) compared with the control animals. These parameters did not differ significantly between the differently treated groups.

**Figure 1:**
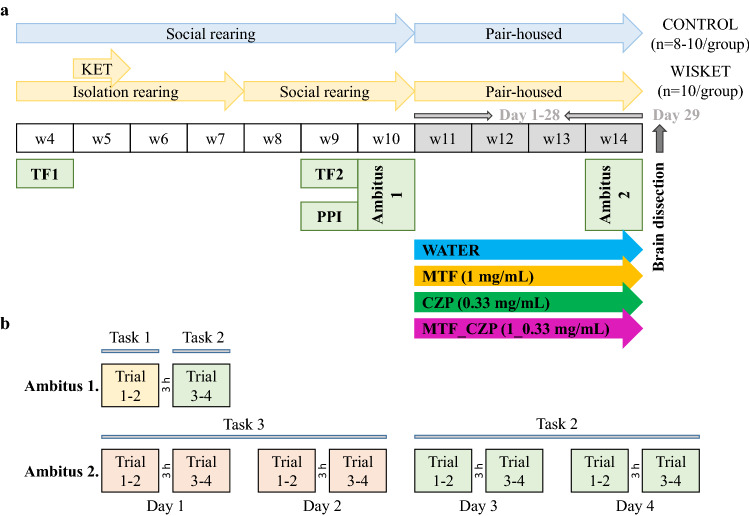
Experimental paradigm (**a**). Abbreviations: w, age in weeks; KET, ketamine; TF, tail-flick test; PPI, pre-pulse inhibition test; MTF, metformin; CZP, clozapine. Experimental paradigm in the Ambitus system (**b**). The Trial 1 and Trial 2 (also Trial 3 and Trial 4) were repeated at intervals of 1 min, with an interval of 3 h between Trial 2 and Trial 3. The means of 4 trials/day were analyzed for the Ambitus test and are referred to as Day 1–4 in Fig. 3. The background color of the trials refers to the Task applied, as follows. Task 1 (yellow): all of the inside and outside boxes were baited (16 rewards); Task 2 (green): the inside boxes alone (8 rewards) were baited; and Task 3 (peach): the outside boxes alone were baited (8 rewards).

**Figure 2:**
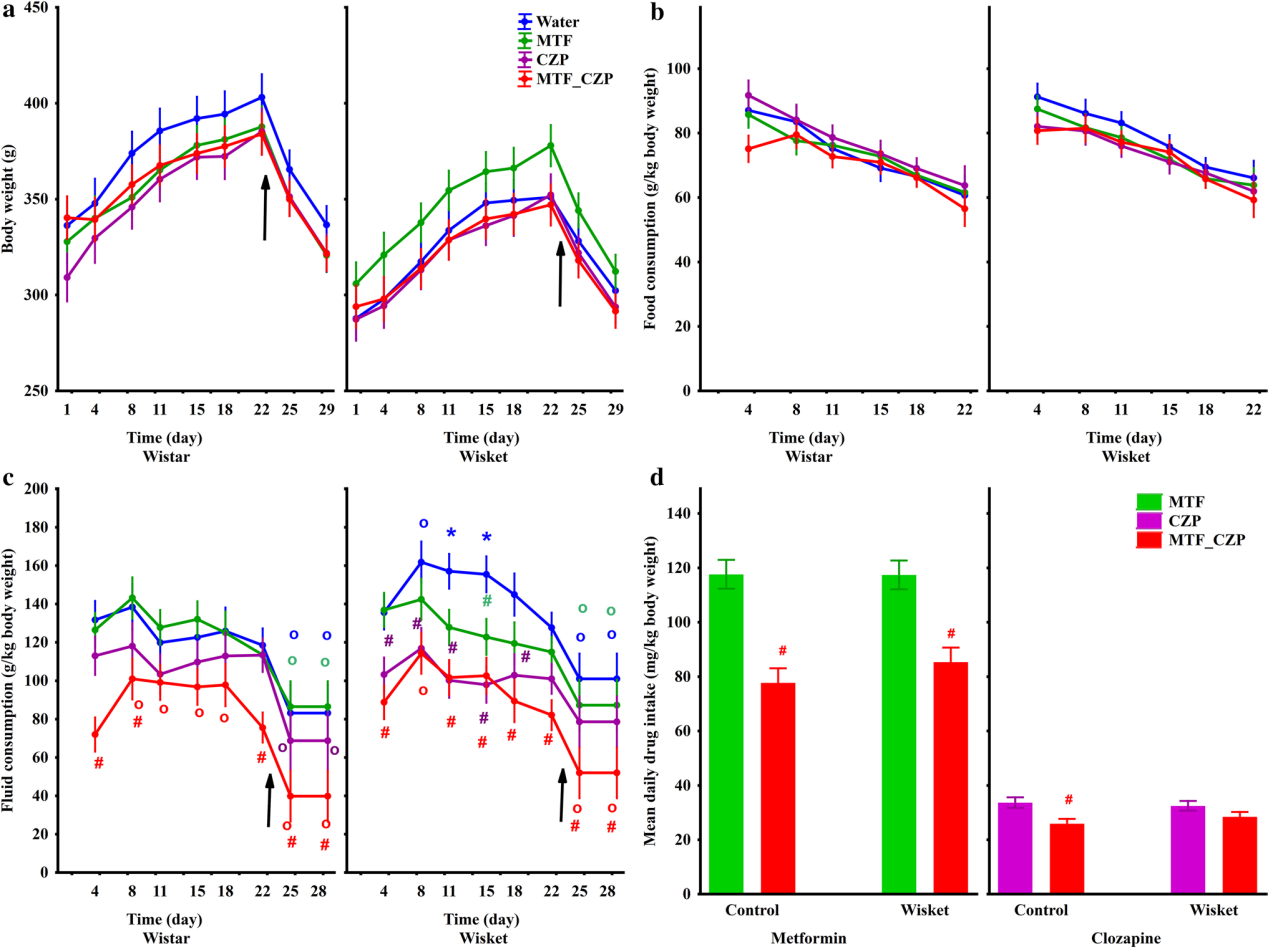
Time-course curves of body weight (**a**), food consumption (**b**), and fluid consumption (**c**) according to group. The symbols indicate significant differences between the Wistar and Wisket groups (*) compared with the water-drinking animals (#), the CZP-alone group (x), and the Day 1 values (o). The arrows indicate the starting of the food restriction. Differences in MTF (left side) and CZP (right side) uptake (**d**) observed between the single and combined treatments during the whole investigation period. The symbol # indicates significant differences between the single and combined treatment groups.

Regarding body weight changes during the treatment period between week 11–14 (Fig. 1), an ANOVA revealed significant effects of group (F_(1,68)_ = 21.27; *P* < 0.001) and time (F_(8,544)_ = 256.76; *P* < 0.001), but not of treatment; i.e., the body weight increased continuously in each group up to the restricted-feeding condition (Day 25, Fig. 2a). Although the Wisket rats had a lower body weight compared with Wistar rats, none of the treatments had significant effects on this parameter. Food consumption was similar in all groups (on days without restriction), but decreased significantly with time (F_(5,150)_ = 61.43; *P* < 0.001; Fig. 2b).

Regarding fluid consumption, this parameter was significantly affected by treatment (F_(3,30)_ = 16.98; *P* < 0.001) and time (F_(7,210)_ = 40.30; *P* < 0.001). The food restriction was accompanied by a decrease in fluid consumption in all groups. The post hoc comparison revealed a significantly higher relative volume of water drinking in Wisket compared with Wistar animals between Days 8 and 15 (Fig. 2c). Furthermore, the control, but not the Wisket, animals treated with the drug combination exhibited a significantly lower fluid intake compared with the animals treated with CZP alone detected on Days 4 and 22. Regarding the analysis of the calculated daily drug intake during the whole investigated period, significant effects of treatments were detected for both MTF and CZP (F_(1,36)_ = 62.60; *P* < 0.001 and F_(1,34)_ = 17.97; *P* < 0.001, respectively; Fig. 2d). The post hoc comparison revealed that the MTF intake was significantly lower in the case of the combination treatment compared with the fluid containing MTF alone in both control and Wisket animals. Furthermore, the CZP intake in the combination treatment was lower compared with the CZP alone in the control, but not in the Wisket, group.

### Behavioral results during the 4-day Ambitus test

**Figure 3:**
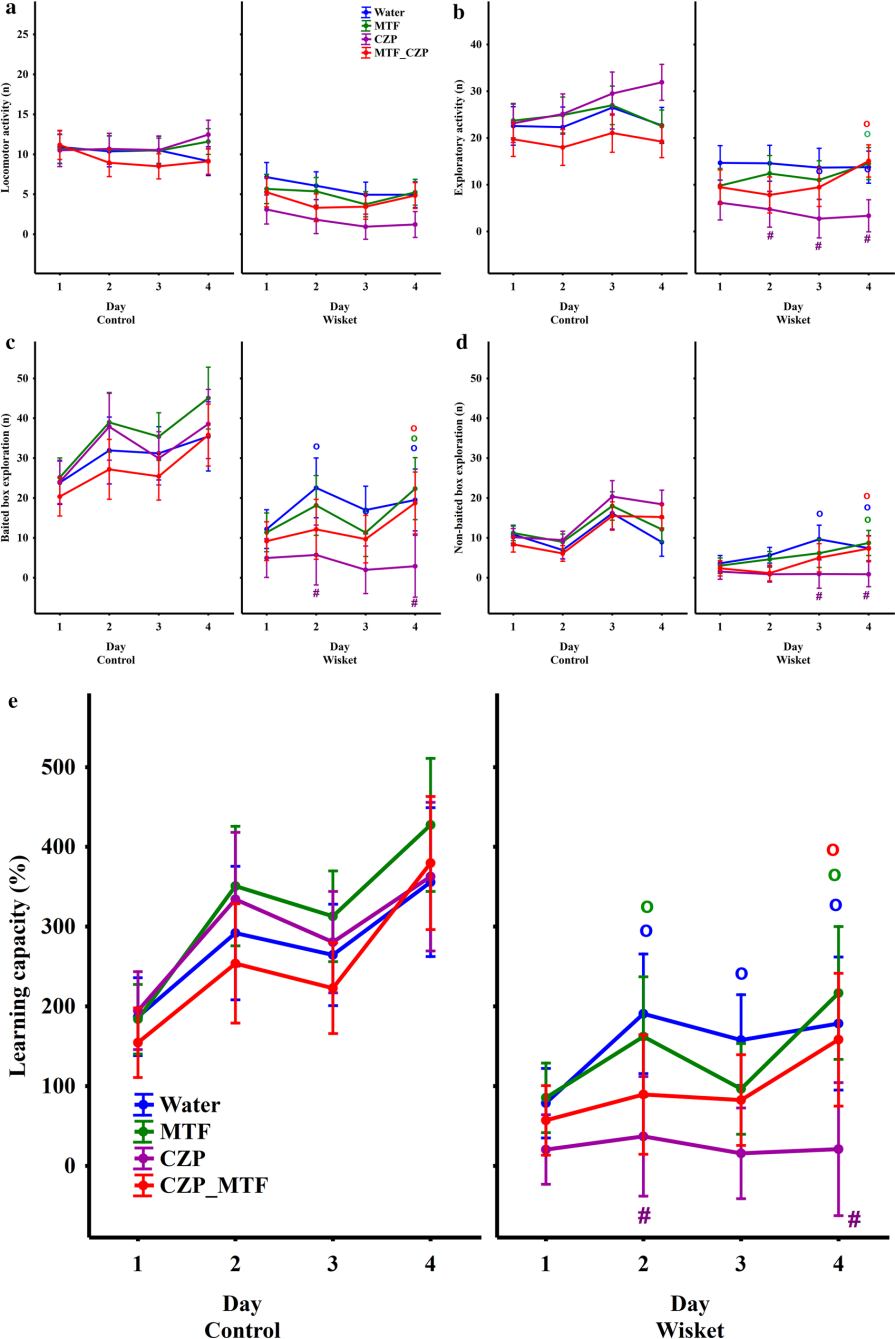
Time-course curves of the locomotor (**a**) and exploratory (**b**–**d**) activities, as well as the learning capacity (**e**), according to group. The symbols indicate significant differences between the Wistar and Wisket groups (*), compared with the water-drinking group (#), and Day 1 values (o).

A repeated measures ANOVA revealed significant effects of group, day, and/or group and day interaction on all of the behavioral parameters assessed here (Table 1). The separate analysis of data from the control group did not reveal significant effects of treatment (Fig. 3). In contrast, significant effects of treatment or time and treatment interaction were detected in the Wisket group for most of the obtained parameters, with the exception of locomotor activity (Table 1). Therefore, only the data of the four Wisket groups were included in the detailed analyses. It was observed that the MTF treatment alone did not produce significant effects on the investigated parameters compared with the water-drinking animals. However, CZP treatment led to a decrease in exploration compared with water-drinking animals, which was accompanied by a decrease in learning capacity (Fig. 3 b–e). All parameters were improved by the MTF_CZP combination treatment, to a level close to that of the water-drinking group.

**Table 1:** ANOVA results for the in vivo measurements.

Parameter	Definition	ANOVA analysis for all groups	Significance: F;(df);p	ANOVA analysis for Wisket groups	Significance: F;(df);p
Locomotor activity	Number of entries into corridors up to 5 min	Group	30.14;(1,68); < 0.001	Treatment	NS
		Day	9.76;(7,476); < 0.001		
Overall exploratory activity	Number of box visits up to 5 min	Group	28.78;(1,68); < 0.001	Treatment/Day	1.75;(7,252); < 0.05
		Day	4.31;(7,476); < 0.001		
		Group/Day	2.1;(7,476); < 0.05		
Baited box exploration	Number of visits into the baited boxes visits up to eating all rewards related to eating time	Group	18.15;(1,68); < 0.001	Treatment/Day	1.72;(7,252); < 0.05
		Day	20.25;(7,476); < 0.001		
		Group/Day	3.13;(7,476); < 0.005		
Non-baited box exploration	Number of visits into the non-baited boxes visits up to eating all rewards related to eating time	Group	22.27;(1,68); < 0.001	Treatment/Day	2.08;(9,108); < 0.05
		Day	21.19;(3,204); < 0.001		
		Group/Day	5.98;(3,204); < 0.001		
Learning capacity (%)	[(eating count)x(300) × 100]/[number of rewards ) × (eating time)]	Group	16.66;(1,68); < 0.001	Treatment/Day	1.72;(21,252); < 0.05
		Day	25.76;(7,476); < 0.001		
		Group/Day	4.32;(7,476); < 0.001

### D_1_R functional activity

**Figure 4:**
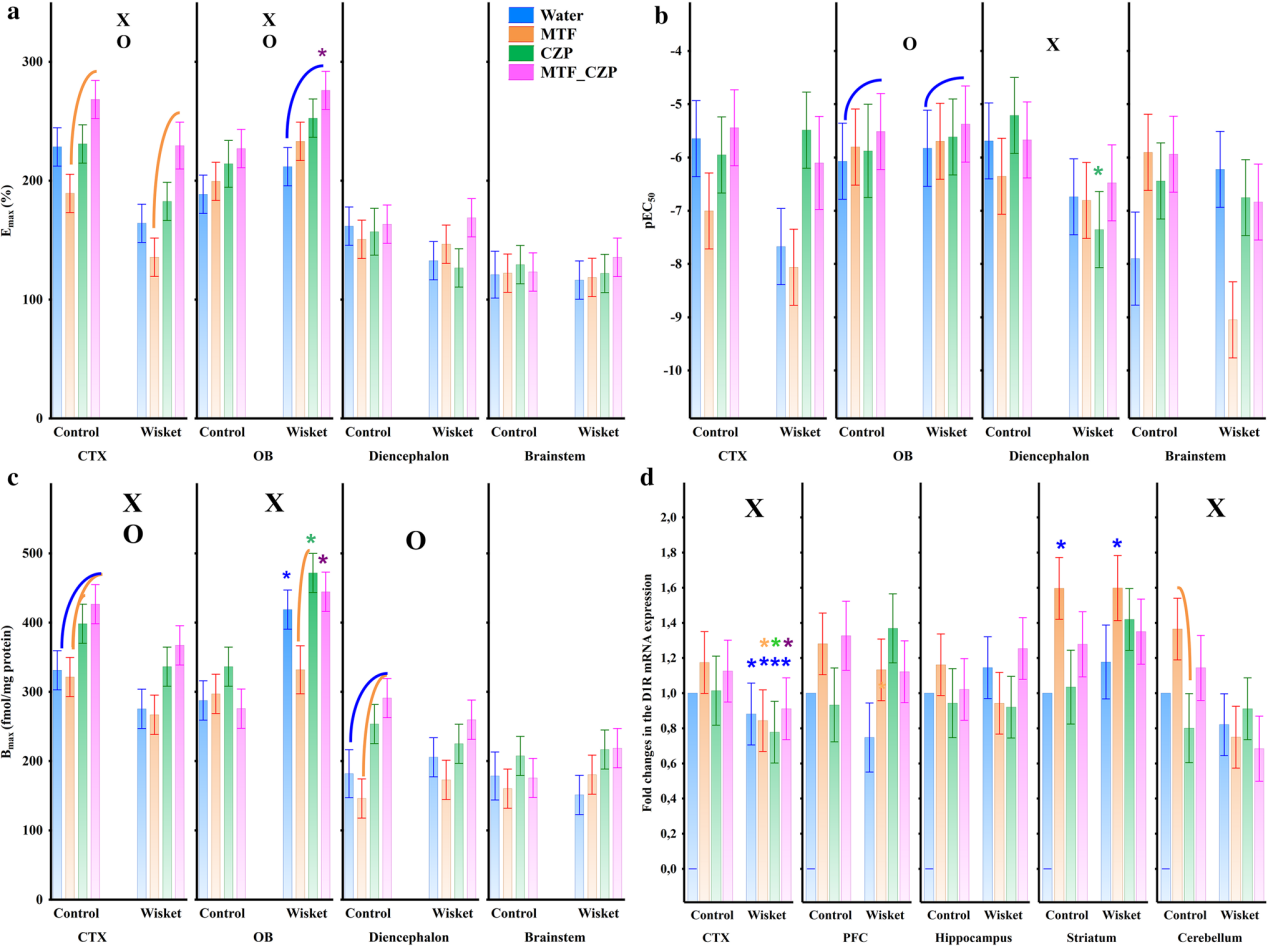
Results of D_1_R signaling, binding, and mRNA expression assays, as indicated by the changes in E_max_ (**a**), pEC_50_ (**b**), and B_max_ (**c**) values and mRNA expression (**d**) in the different rat brain structures. The symbols indicate significant effects of group (X) and treatment (O). The symbols also indicate post hoc significant differences between groups (*) and treatments (arc). The blue stars pinpoint significant differences compared with the water-drinking Wistar rats, whereas the remaining stars indicate significant differences compared with the control matched pairs. Abbreviations: CTX, cerebral cortex; OB, olfactory bulb; PFC, prefrontal cortex.

In the [^35^S]GTPγS binding assays, the maximal stimulation (efficacy [E_max_]) of G-protein and the negative logarithm of ligand potency (pEC_50_) were determined after agonist occupation of the D_1_Rs. Regarding the G-protein activation in the CTX, the E_max_ values revealed significant effects of group (F_(1,15)_ = 8.17; *P* < 0.05) and treatment (F_(3,15)_ = 3.71; *P* < 0.05; Fig. 4a). The post hoc analysis showed that both MTF_CZP groups had a significantly higher E_max_ level compared with their MTF-treated matched pairs. The E_max_ values also showed significant effects of group (F_(1,15)_ = 12.57; *P* < 0.005) and treatment (F_(3,15)_ = 4.97; *P* < 0.05; Fig. 4a) in the OB. The post hoc comparison revealed that the Wisket animals treated with the MTF_CZP combination had a significantly higher level of maximal G-protein activation compared with their matched control animals and to water-drinking Wisket animals. The brainstem and diencephalon were not affected by any of the treatments in this respect. Regarding the pEC_50_ values (Fig. 4b), a significant effect of treatment was detected in the OB (F_(3,15)_ = 4.82; *P* < 0.05), and the post hoc comparison revealed that the MTF_CZP-treated groups had higher values compared with their water-drinking counterparts. Furthermore, the diencephalon exhibited a significant effect of group on this parameter (F_(1,16)_ = 6.95; *P* < 0.05), with lower values detected in Wisket animals. No significant effects were observed in the brainstem.

In equilibrium-saturation-binding assays, the maximal number of specific radioligand binding sites (capacity, B_max_) and the affinity of the ligand–receptor interaction (dissociation constant [K_d_]) were established. According to the saturation-binding experiments, significant effects of group (F_(1,16)_ = 12.77; *P* < 0.005) and treatment (F_(3,16)_ = 9.42; *P* < 0.05; Fig. 4c) on the B_max_ values of the CTX were detected, with a lower level of binding observed in the Wisket animals. The post hoc comparison uncovered significantly higher values in the MTF_CZP Wistar group compared with its water- or MTF-drinking counterparts, and the same trend was detected in the Wisket rats. The ANOVA of B_max_ values in the OB revealed significant effects of group (F_(1,15)_ = 20.22; *P* < 0.001), with a higher level detected in the Wisket animals. The post hoc comparison showed significant differences between the control and Wisket animals for the water, CZP, or MTF_CZP treatments, with significantly lower B_max_ values detected in the MTF- compared with CZP-treated Wisket animals (Fig. 4c). Regarding the diencephalon, significant effects of treatment (F_(3,15)_ = 5.20; *P* < 0.05) were observed, i.e. significantly higher binding capacity was detected in the MTF_CZP-treated Wistar group compared with water- or MTF-drinking animals. No significant effects were observed in the brainstem. The K_d_ values were not significantly affected by any of the treatments (data not shown).

### D_1_R mRNA expression

The factorial ANOVA of relative mRNA expression values revealed significant effects of group in the CTX (F_(1,68)_ = 22.04; *P* < 0.001) and cerebellum (F_(1,66)_ = 5.05; *P* < 0.05), with a lower level detected in the Wisket animals (Fig. 4d). The post hoc comparison showed that the D_1_R mRNA expression was significantly lower in the cerebellum of the MTF-treated Wisket animals compared with their matched controls. An unpaired *t*-test revealed that the MTF treatment increased the D_1_R mRNA expression in the striatum in both Wistar and Wisket animals compared with their water-drinking counterparts. No significant effects were observed in the PFC and hippocampus.

## Discussion

This study characterized the effects of chronic treatment with MTF, CZP, and their combination on several behavioral parameters (motor activity and learning function) and D_1_R activation, binding, and gene expression in different brain structures. It was revealed that none of these treatments significantly affected the behavioral profile of control animals. In contrast, CZP treatment caused further impairments in the exploratory activities and cognitive functions in Wisket animals compared with their water-drinking counterparts. Although MTF alone did not significantly affect these parameters, it blunted the behavioral side effects of CZP. The in vitro results showed a region-specific alteration in D_1_R expression and/or signaling in several brain regions of the Wisket animals, which were influenced primarily by the combination treatment.

Cognitive dysfunction remains an unresolved problem in the successful management of schizophrenia. It is well known that this phenomenon is highly dependent on behavioral activity, attention, and motivation, which are also impaired in these patients^24–26^. Conflicting results are available regarding the efficacy of CZP against cognitive symptoms in patients with schizophrenia^3,14–17^. Furthermore, atypical antipsychotic treatment is often complicated by the development of obesity and diabetes, leading to metabolic syndrome, and these factors have also been linked to the increased risk of cognitive impairments^15,27^. In agreement with these results, it is very important to consider that the second-generation antipsychotics, including olanzapine and CZP, may result in sustained hyperglycemia when administered to rats chronically, which can cause detrimental changes in the brain and might affect behavior^28,29^. These effects are prevented by regulating glucose levels through exercise. Of relevance to this study, MTF has also been shown to reverse antipsychotic-induced glucose dysregulation in rats^30^. Therefore, the well-known effect of MTF on blood glucose levels may be a very important factor that might be involved in the beneficial cognitive effects of the co-administration of MTF with a second-generation antipsychotic drug. CZP, as many other psychoactive drugs, decreases the activity and operant response performance for food reward, which might be attributed to its sedative effects, at least partially, leading to impairments in cognitive functions^16,17^. In agreement with these results, CZP significantly decreased the exploratory activity in the Wisket animals, suggesting a blunted motivation. MTF, which is a first-line agent in the treatment of patients with type II diabetes mellitus, can rapidly cross the blood–brain barrier and exert a protective effect on cognitive functions^7,8,31^. Although patients with schizophrenia are frequently treated with MTF to blunt the antipsychotic-induced metabolic side effects, its effect on their cognition has not been evaluated. Only two preclinical studies have investigated the behavioral effects of MTF in schizophrenia models^19,20^. In a rat model induced by the NMDA receptor antagonist MK-801, MTF significantly ameliorated the memory impairments in the water maze test, but had no effect on the basic anxiety-like levels in normal naive rats^19^. In contrast to these data, the cognitive function of Wisket rats was not affected significantly by treatment with MTF alone; this difference might be attributed to the altered type of schizophrenia model and/or the applied behavioral tests (reward- vs. punishment-based tests). Using a mouse model incorporating both MK801 and chronic unpredictable mild stress exposures, the signs of psychosis and depression were replicated^20^. The administration of triple-drug combinations consisting of two antidepressants plus CZP improved the performance of these animals in behavioral assays. Moreover, the addition of MTF to the treatments further improved both the depressive and schizophrenia-like behaviors. However, this study did not investigate the cognitive function of these animals, and the combination of the four drugs might inhibit the determination of the interaction between CZP and MTF. Our results clearly showed that the CZP-induced behavioral impairments were effectively improved by MTF co-administration.

There is strong supportive evidence for the role of D_1_Rs in cognitive functions, and an insufficient D_1_R function has been linked to working memory impairments, at least partly by affecting the reward mechanism^32–34^. D_1_Rs are widely distributed in the human, primate, and rodent brain, with the highest levels detected in the striatum, CTX, and hippocampus; moreover, these receptors can be found in the brainstem, OB, cerebellum, and diencephalon (hypothalamus and thalamus)^35–39^. In agreement with these data, the presence of D_1_Rs could be detected in all of the investigated areas, with a high level of receptor density (B_max_) and increased G-protein activation (E_max_) observed in the CTX. The D_1_R has now been highlighted as an important neurobiological target for the treatment of schizophrenia^1,40^. Neuroimaging studies in schizophrenia have reported conflicting results regarding D_1_R density and/or activation in the brain^39,41–43^. Thus, in vivo imaging studies of dopaminergic neurotransmission in acute schizophrenia have confirmed the upregulation of D_1_Rs in the striatum and CTX, with no changes detected in the thalamus, temporal cortex, and hippocampus^43^. However, other studies found a decreased D_1_R-binding potential in the frontal cortex of patients with schizophrenia; alternatively, they did not find any changes in different cortical areas^39,41,42^. There is a large disagreement about the effects of CZP on the D_1_Rs, i.e., it has been mentioned as an agonist, antagonist, or inverse agonist ligand^33,44^. Furthermore, CZP has affinity for other neurotransmitter systems (e.g., serotoninergic, histaminergic, adrenergic, and cholinergic) implicated in attention, motivation, and/or sedation,; therefore, its disruptive effects may result from combined effects on them, leading to depressed spontaneous activity and impaired reward and cognitive functions^33,45^. Few studies have suggested a connection between MTF and the dopaminergic system; MTF prevented nigrostriatal dopamine degeneration and attenuated the development of dyskinesia, but did not affect downstream mediators of D_1_R hyperactivation in the striatum in models of Parkinson’s disease^8,46^.

Our data showed that the most obvious changes occurred in the CTX; the Wisket animals had significantly lower gene expression, binding capacity, and G-protein activation, suggesting significant impairments in the cortical D_1_R function in this schizophrenia animal model. In contrast, previous data revealed that the chronic administration of CZP increased D_1_R mRNA expression moderately in the rat cortex^37^, whereas none of the treatments affected this process significantly in our experiments. However, the combined treatment significantly enhanced both the E_max_ and B_max_ values in this area, which might play role in the beneficial effects of the combined treatment regarding the behavior of Wisket animals.

The OB is rich in neurons containing both GABA and dopamine, and both D_1_Rs and D_2_Rs are expressed in this brain region^38,47^. It also plays a significant role in cognitive processes, which might be provided, at least partially, through D_1_Rs^38,48^. Surprisingly, both the D_1_R binding level and G-protein-mediated transmembrane signaling in the OB were higher in Wisket animals, suggesting an increased D_1_R density in this structure. Furthermore, the combined treatment caused a significant enhancement in the efficacy of G-protein activation in these animals, and a similar trend was detected in the control animals, which was accompanied by a lower level of ligand potency. It cannot be excluded that these effects might also play role in the beneficial effects of combined treatments observed in the Wisket group.

The activation of D_1_Rs in different nuclei of the brainstem and diencephalon may be involved in the regulation of multiple physiological functions (e.g., feeding, pain sensation, sensory gating, and circadian rhythm), which are also disturbed in patients with schizophrenia^36,49^. No changes were observed in the brainstem, whereas a higher ligand potency (i.e., lower pEC_50_ values) was detected in the diencephalon of Wisket animals. Furthermore, the combination-treatment-induced enhancement of the B_max_, without changes in the E_max_ values, observed mainly in the control animals, suggests that the enhanced density of binding sites was accompanied by a decreased G-protein activation in the diencephalon.

Regarding the region-specific mRNA expression of D_1_Rs, clinical data have revealed decreased expression of the D_1_R transcript in the PFC of patients with schizophrenia, whereas the hippocampus and caudate nucleus did not exhibit alterations^32,50^. Furthermore, subchronic exposure to an NMDA receptor antagonist (as a schizophrenia model) downregulated the D_1_R mRNA in the PFC^21^, similar to the tendency found here in Wisket rats. Only one study showed that subchronic treatment with CZP did not reverse the decrease in frontal cortex D_1_R density, as assessed using post-weaning isolation rearing; rather, it increased its affinity^22^. In contrast, we did not find any effects of CZP treatment on D_1_R mRNA expression, which might be attributed to differences in the models and/or the treatment paradigms.

In agreement with earlier data, no significant differences were observed between the two groups regarding D_1_R mRNA expression in the striatum and hippocampus; moreover, chronic administration of CZP did not modify this process in rodents^37,39,50^. However, MTF administration alone caused a significant increase in D_1_R expression in the striatum in both groups, without any consequence for the behavioral parameters. In addition to motor coordination, the cerebellum is also well known for its role in cognition, and schizophrenia is associated with alterations in cerebellar function^51,52^. Consistent with this role, the decreased D_1_R mRNA expression detected in the Wisket animals may also be involved in their behavioral impairments.

In summary, the in vitro data revealed an impairment of the D_1_R system in the Wisket animals; moreover, the CZP treatment alone did not significantly modify any of the in vitro parameters in the two groups. In contrast, the combined treatment had significant effects in several brain structures in both groups in the signaling and binding experiments; primarily, it enhanced the maximal G-protein activation and maximal D_1_R binding in the CTX in both groups. Although the changes in the D_1_R functions evoked by the combination therapy were not accompanied by behavioral alterations in the control animals, it can not be excluded that the improvement in D_1_R function observed in the Wisket animals stemmed from the beneficial effects of the drug combination. As discussed above, the two drugs can influence several other systems that are involved in cognitive functions; thus, the complex interaction between the different transmitter systems might have led to the beneficial effects of this combination observed in the Wisket animals.

## Limitations

Our work needs to be interpreted with caution, for several reasons. First, it should be mentioned that, in this paradigm, we applied a shorter period of ketamine treatment compared with our earlier studies (5 vs. 15 days), to protect the animals from the severe side effects of prolonged treatment (e.g., diarrhea)^9,10^. However, the preservation of significant differences between the control and Wisket rats in the behavioral tests suggests that the model rats also have a schizophrenia-like phenotype in this condition.

As in earlier studies, while applying the drinking water drug-intake method, the rats were housed in pairs to avoid the stress of social isolation; therefore, it was not possible to accurately determine the food and fluid intake and drug doses in the individual animals^46,53^. However, none of the treatments significantly affected the body weight of the animals.

Another possible confounding bias of our study is that the amount of drinking water affected the fluid consumption, and it seems that the MTF_CZP combination was distasteful for the animals. This effect was prominent in the control rats; however, the lower level of fluid intake did not cause any signs of behavioral impairment. A similar trend was detected in the Wisket animals, but it did not reach a significant level, and neither body weight nor food consumption were affected by this treatment. Furthermore, the post hoc analysis revealed the absence of significant differences in fluid intake between the CZP- and combination-treated Wisket animals at any of the investigated time points. Despite the lower fluid intake observed in the MTF_CLZ groups, the doses of the CZP and MTF intake were in agreement with previous studies^8,15^. Thus, even lower doses of CZP (1–10 mg/kg/day) or MTF (50 mg/kg) produced effects on cognitive functions^15,16^. Therefore, we concluded that the beneficial behavioral effects of the combined treatment can be attributed to MTF, rather than the slightly lower level of CZP intake.

Since both CZP and MTF have significant effect on carbohydrate metabolism^54^, a major limitation of this study is that the glucose metabolism was not investigated. Thus it can not be excluded that the interaction of MTF and CZP on metabolic parameters (e.g. through glucagon-like peptide regulation) might also contribute to the enhanced cognition that we observed^55^. Therefore, further studies are needed to determine the glucose metabolism in Wisket animals in this experimental paradigm to provide valuable information about the antipsychotic-induced metabolic syndrome and its improvement by an antidiabetic drug.

It is perfectly clear that a correlation analysis between the behaviors and the D_1_R system would have provided valuable data about the relationship between them. During the behavioral studies, it was possible to test the experimental animals individually. In in vitro measurements, however, we combined tissue samples from several animals belonging to the same experimental group, especially during radioligand binding tests. There were two main reasons for this approach: (1) the large number of test samples necessary for determining the concentration dependence of the ligand binding parameters over a wide range and (2) the requirement for a protein content of the cell membrane fractions used in the receptor binding experiments of at least 100 µg in each reaction tube. To fulfill these two conditions, we were forced to combine samples from the brain areas of different animals. Combining tissue samples from different animals in this way is common practice in experiments carried out for biochemical purposes^56^. The preparation procedure adopted here allowed the relatively accurate determination of each measurement parameter. Therefore, our data are suitable for the comparison of receptor function or mRNA expression in various brain areas; however, in our opinion, they cannot be directly compared with data from the in vivo studies. For these reasons, we could not perform such a correlation analysis.

## Conclusion

Taken together, the results obtained here demonstrates that CZP, MTF, and their combination did not affect behavioral parameters in control animals in a reward-based learning paradigm. In contrast, CZP caused further impairments in Wisket animals, an effect that was blunted by MTF co-treatment. Because patients with schizophrenia are frequently treated with MTF to decrease the metabolic side effects of antipsychotics, the effects of MTF on cognition in this patient group should also be determined. In agreement with human studies, the cortical D_1_R mRNA expression, binding, or signaling was decreased in the Wisket animals and was affected primarily by the combined treatment with CZP and MTF.

## Methods

### Animals

Male Wistar (control) and Wisket rats were included in this study. All experiments were carried out with the approval of the Hungarian Ethical Committee for Animal Research (registration number: XIV/1248/2018) and in accordance with the guidelines set by the Government of Hungary and EU Directive 2010/63EU for animal experiments. It is confirmed that the study was carried out in compliance with the ARRIVE guidelines. Animals were kept under a 12 h light/dark cycle with conditions of controlled temperature (22 °C ± 1 °C) and humidity (55% ± 10%). The behavioral experiments were performed between 8 a.m. and 4 p.m., under dim lighting. The body weight of the rats was carefully controlled during the whole experiment.

### Experimental paradigm

Based on our earlier studies, after weaning (on week 4), both control and Wisket rats were tested in the tail-flick test (48 °C hot water) to assess their basal acute heat pain sensitivity (Fig. 1a)^9,10^. Subsequently, the Wisket animals were housed individually for 28 days and treated with ketamine (30 mg/kg/day intraperitoneally, for 5 days) during the 2^nd^ week of isolation rearing (Calypsol, Gedeon Richter Plc., Budapest, Hungary). The animals were then re-housed (3–4 animals/cage), followed by 1 week of recovery with no treatment. During this period, control animals were socially reared (3–4 animals/cage), with no ketamine treatment.

On week 9, all animals were included in tail-flick and sensory gating (pre-pulse inhibition: PPI) tests. The PPI of the acoustic startle response was measured in the Startle and Fear Conditioning System (Panlab, S.L.\Harvard Apparatus; Barcelona, Spain) after 12 h of food withdrawal. After a 7.5-min habituation in startle chambers using a background noise of 60 dB, rats were exposed to two different trial types: the pulse alone (PA), in which a 40 ms, 115 dB white noise burst was presented; and the prepulse–pulse pair (PP), in which prepulse stimuli (20 ms, 85 dB) were followed by the startle stimulus with a latency of 150 ms. Both types of stimuli were applied 20 times using a random pattern. The interstimulus intervals ranged from 7 to 13 s. The PPI was calculated as a percentage using the following equation: PPI (%) = [1 − (startle response for PP) /(startle response for PA)] × 100. While more reliable data could have been detected on sensory gating properties by applying multiple different prepulse intensities, our preliminary experiments showed that the prolonged detection of startle response led to the inactivity of the animals.

On week 10, the basal locomotor and exploratory activities and cognitive function were assessed in the AMBITUS 2–2 trials using Task 1 and Task 2 (Ambitus 1, see below and Fig. 1).

On the following week, 4–4 groups of control and Wisket rats were included in a 28-day experiment based on the content of the drinking fluid: water, MTF, CZP, or MTF_CZP combination (Fig. 1b). CZP and MTF were dissolved and diluted with water. The animals were assigned to the pharmacological treatment groups based on their basal test results and body weight to be identical to each other within the control and Wisket groups. The oral route (drinking water) was chosen for the administration of the compounds, as it is the preferred translational option for potential use in humans. Although daily gavage might represent a more reliable method of substance administration, we aimed to avoid the repeated stress it would cause over this extended period (28 days). The concentration of MTF was 1 mg/mL, whereas that of CZP was 0.33 mg/mL in the fluid of both the single and combined drug-treated groups. The dosing strategy was based on previously published rodent studies^8,15^. The animals were pair-housed for the whole experimental paradigm, as described previously^46,53^. Although this paradigm precluded the precise measurements of fluid and food consumption of the individual animals, we aimed to avoid the effects of social isolation. The body weight and relative food and fluid consumption were determined twice a week during the experiment and freshly prepared solutions were provided. Because restricted food availability was applied 2 days before and during the Ambitus test (see below), the degree of food consumption was analyzed only at the phases during which food was freely available.

A 4-day Ambitus test (Ambitus 2) was performed on week 4 of the drug treatments by applying Task 3 and Task 2 (see below and Fig. 1b) after a 2-day food-deprivation period. Moderate food restriction was maintained throughout the Ambitus test by decreasing the amount of food (10–15 g/day). Drinking fluid was freely available during the whole experiment.

### The AMBITUS apparatus

The Ambitus apparatus is a rectangular corridor constructed of clear plexiglass on a black floor with an external diameter of 80 cm, a width of 8 cm, and a height of 50 cm (www.deakdelta.hu), where the rats could move around forward and backward between the walls^10^. Each of the four corridors has four side-boxes with an equal size (5 × 5 × 5 cm; 2–2 on the internal and external walls; altogether, 16) with a food reward (puffed rice: 20 mg). Infrared beams located at the entrance of each aperture allowed the detection of nose pokes (exploration), whereas the locomotor activity was detected by infrared beams located midway in each corridor, at a 1 ms time resolution.

After the insertion of the food rewards into the side boxes, trials commenced by placing the rats at the same starting point within the corridor; thereafter, the experimenter immediately left the room^10^. The animals were allowed to explore the corridor and collect food rewards for 5 min (cut-off time: 300 s). At the end of each trial, the number of food rewards eaten by the animals was recorded and the apparatus was cleaned with 70% ethanol. Experiments were recorded using an infrared video device (WCM-21VF, CNB, China) fastened above the apparatus. If an animal had eaten all the available rewards, its video recording was analyzed offline to determine the time required to complete the task. Three different tasks were applied during the study (Fig. 1b). In Task 1 (trials 1 and 2 during the baseline measurements), all inside and outside boxes were baited (16 rewards); in Task 2 (trials 3 and 4 during the baseline measurements and Days 3–4 during the 4-day-long experiment), only the inside boxes (8 rewards) were baited; whereas in Task 3 (Days 1–2 during the 4-day-long experiment), only the outside boxes were baited (8 rewards). All rats performed two sessions (two trials/session, 1 min apart) of tasks per day, one in the morning and another about 3 h later (4 trials/day). Altogether, 16 trials were completed, and the means of the daily 4 trials were further analyzed. Table 1 shows the definition of the analyzed parameters in the Ambitus system.

### Brain extraction

One day after conducting the Ambitus 2 test (Day 29, Fig. 1a) the animals were decapitated and the brains were quickly removed, dissected on dry ice, frozen in liquid nitrogen and stored at − 80 °C until further analyses.

### Preparation of brain samples for the binding assays

Neuronal membrane fractions were prepared from frozen brains for in vitro receptor binding and the functional [^35^S]GTPγS (specific activity: 1000 Ci/mmol; purchased from Hartmann Analytic, Braunschweig, Germany) binding experiments, according to our previous studies^12,57^. The protein content of the membrane preparation was determined via the method of Bradford using BSA as a standard^58^, and the UltimaGold MV scintillation reagent was from PerkinElmer (Boston, USA).

### Functional [^35^S]GTPγS binding experiments

The functional [^35^S]GTPγS binding experiments were performed as previously described^59,60^, with modifications for optimizing the binding assay stimulated with a D_1_R agonist^61^. Briefly the membrane homogenates were incubated at 30 °C for 60 min in buffer (pH 7.4) composed of 25 mM HEPES, 120 mM NaCl, 20 mM MgCl_2_, 1.8 mM KCl, and 1 mM sodium deoxycholate containing 20 MBq/0.05 cm^3^ [^35^S]GTPγS (0.05 nM) and increasing concentrations (10^—^^10^ to 10^—^^5^M) of the selective D_1_R full agonist, SKF81297 (Tocris Bioscience, Bristol, United Kingdom)^62^. The experiments were performed in the presence of excess GDP (10 µM) in a final volume of 1 mL. Total binding was measured in the absence of test compounds, whereas non-specific binding was determined in the presence of 10 µM unlabeled GTPγS and subtracted from the total binding. The difference represented basal activity. The reaction was terminated by rapid filtration under vacuum (Brandel M24R Cell Harvester), and washed three times with 5 mL of ice-cold 0.1 M phosphate (pH 7.4) buffer through Whatman GF/B glass fibers. The radioactivity of the dried filters was detected in an UltimaGold MV aqueous scintillation cocktail on a Packard Tricarb 2300TR liquid scintillation counter. [^35^S]GTPγS binding experiments were performed in triplicate and were repeated at least three times.

### Saturation-binding experiments

Aliquots of frozen rat brain membrane homogenates were centrifuged, thawed, and suspended in 50 mM Tris–HCl buffer (pH 7.4). Because the D_1_R antagonist [^3^H]SCH 23,390 (Tocris Bioscience, Bristol, United Kingdom) also possesses high affinity for the 5-HT_2_ receptors^63^, 1 µM ketanserin (a selective 5-HT_2_ ligand) was added to the buffer, to block them^64^. Membranes were incubated in the presence of [^3^H]SCH 23,390 in increasing concentrations (0.29–12.01 nM) at 25 °C for 60 min. The non-specific and total binding were determined in the presence and absence of 10 µM unlabeled SCH 23,390, respectively. The reaction was terminated by rapid filtration under vacuum (Brandel M24R Cell Harvester) and washed three times with 5 ml of ice-cold 50 mM Tris–HCl buffer (pH 7.4) through Whatman GF/B glass fibers. The radioactivity of the dried filters was detected in an UltimaGold MV aqueous scintillation cocktail on a Packard Tricarb 2300TR liquid scintillation counter. The saturation-binding assays were performed in duplicate and were repeated at least three times.

### Gene expression analysis

RNA extraction and quantitative real-time polymerase chain reaction (qRT-PCR).

Total RNA was extracted from tissue samples that were homogenized in TriXtract reagent (G-Biosciences, St. Louis, MO, USA). Subsequently, the RNA was separated into an aqueous phase by adding chloroform. The RNA pellet was dissolved in RNAse-free water after precipitation with isopropyl alcohol and a wash with 70% ethanol. The quantity and quality of the extracted RNA were checked using a Genova Nano micro-volume spectrophotometer (Jenway) at an optical density of 260 and 260/280 nm, respectively. The samples used for further analyses exhibited an absorbance ratio in the range of 1.6–2.0. Equal amounts of RNA were employed to synthetize cDNA in each experiment using the iScript cDNA synthesis kit (Bio-Rad, Hercules, CA, USA).

PCR was carried out in a thermo cycler (Bio-Rad CFX96 Optics Module) by preparing triplicates of reactions of 10 µl using the iQ SYBR Green Supermix (Bio-Rad). The thermal cycling conditions included an initial denaturation step at 95 °C for 30 s and 40 cycles of denaturation at 95 °C for 10 s, annealing at 59 °C for 30 s, and extension at 72 °C for 20 s. The amplicons were subjected to a melting curve analysis. The pair of primers previously designed by Bangaru et al. was applied in-house to identify glyceraldehyde 3-phosphate dehydrogenase (GAPDH) as the endogenous control (forward: 5′–AAGAAGGTGGTGAAGCAGGCG–3′ and reverse: 5′–AGCAATGCCAGCCCCAGCAT–3′)^65^. Two primer pairs were designed using the National Centre of Biotechnology Information reference sequence database (https://www.ncbi.nlm.nih.gov/Entrez) to amplify a 92 bp fragment of the D_1_R mRNA (forward: 5′-GATCTCTTGGTGGCTGTCCTG-3′ and reverse: 5′-ACCCAGATGTTACAAAAGGGAC-3′). In the negative controls, the reaction contained RNAse-free water instead of cDNA. The threshold cycle (Ct) values were used as reference points and the comparative Ct method (Δ∆Ct method) was implemented to achieve relative quantification^66^; 2^−Δ∆Ct^ values were used to calculate fold changes in target gene expression using the control groups as normalizers.

### Statistical analysis

All data are expressed as means ± S.E.M., and significance was set at *P* < 0.05 level. For the statistical analyses, the STATISTICA 13.4.0.14 (TIBCO Software Inc., USA) and GraphPad Prism (Inc., San Diego, CA) software were used.

For in vivo experiments, data were evaluated by factorial or repeated measures ANOVA, where the repeated measures were days and the factors were group (control, WISKET) and treatment (water, MTF, CZP, and MTF/CZP). Table 1 provides definitions and denotes the significances of the analyzed behavioral parameters. Post hoc comparisons were performed using Fisher’s LSD test.

The radioreceptor binding (saturation curves, one binding site model) and [^35^S]GTPγS binding data (sigmoid dose response stimulation) were processed by a professional curve-fitting program (GraphPad Prism 5.0.) using a non-linear regression analysis. A factorial variance analysis was performed to determine the significance level of groups and treatments for the obtained in vitro parameters. The post hoc comparisons were performed by using the Fisher LSD test. For the mRNA expression data, the unpaired t-test was also used to determine the differences between the different groups compared to the water-drinking control animals.
